# Data Visualization Preferences in Remote Measurement Technology for Individuals Living With Depression, Epilepsy, and Multiple Sclerosis: Qualitative Study

**DOI:** 10.2196/43954

**Published:** 2024-10-18

**Authors:** Sara Simblett, Erin Dawe-Lane, Gina Gilpin, Daniel Morris, Katie White, Sinan Erturk, Julie Devonshire, Simon Lees, Spyridon Zormpas, Ashley Polhemus, Gergely Temesi, Nicholas Cummins, Matthew Hotopf, Til Wykes, RADAR-CNS Consortium

**Affiliations:** 1 Institute of Psychiatry, Psychology and Neuroscience King's College London London United Kingdom; 2 Remote Assessment of Disease And Relapse - Central Nervous System Patient Advisory Board King's College London London United Kingdom; 3 Medical Science Division IT Services Prague Czech Republic; 4 South London and Maudsley NHS Foundation Trust London United Kingdom

**Keywords:** mHealth, qualitative, technology, depression, epilepsy, multiple sclerosis, wearables, devices, smartphone apps, application, feedback, users, data, data visualization, mobile phone

## Abstract

**Background:**

Remote measurement technology (RMT) involves the use of wearable devices and smartphone apps to measure health outcomes in everyday life. RMT with feedback in the form of data visual representations can facilitate self-management of chronic health conditions, promote health care engagement, and present opportunities for intervention. Studies to date focus broadly on multiple dimensions of service users’ design preferences and RMT user experiences (eg, health variables of perceived importance and perceived quality of medical advice provided) as opposed to data visualization preferences.

**Objective:**

This study aims to explore data visualization preferences and priorities in RMT, with individuals living with depression, those with epilepsy, and those with multiple sclerosis (MS).

**Methods:**

A triangulated qualitative study comparing and thematically synthesizing focus group discussions with user reviews of existing self-management apps and a systematic review of RMT data visualization preferences. A total of 45 people participated in 6 focus groups across the 3 health conditions (depression, n=17; epilepsy, n=11; and MS, n=17).

**Results:**

Thematic analysis validated a major theme around design preferences and recommendations and identified a further four minor themes: (1) data reporting, (2) impact of visualization, (3) moderators of visualization preferences, and (4) system-related factors and features.

**Conclusions:**

When used effectively, data visualizations are valuable, engaging components of RMT. Easy to use and intuitive data visualization design was lauded by individuals with neurological and psychiatric conditions. Apps design needs to consider the unique requirements of service users. Overall, this study offers RMT developers a comprehensive outline of the data visualization preferences of individuals living with depression, epilepsy, and MS.

## Introduction

Remote measurement technology (RMT) involves the use of wearable devices and smartphone apps to measure health outcomes in everyday life. Real-time, continuous symptom tracking has the potential to revolutionize the self-management of chronic conditions [1]. Feedback of data, in the form of data visual representations, can facilitate patient-driven health care and present opportunities for intervention by raising awareness of symptom patterns or prompting health appointments [2-5]. There is an emerging literature on “data visualization,” which is defined as “the visual representation of data, encoded using position, length, size, and or color, among others, to reduce complexity and effectively communicate information to support discovery and understanding of patterns within data, decision-making, and memory” [6].

RMT users living with neurological and psychiatric conditions consistently express a desire to visualize their recorded data [7-9]. This includes both condition-specific metrics, as well as measures of broader health such as physical activity and sleep patterns. This could help to increase RMT users’ engagement with the recording technology [10,11], improve communication with others involved in their care [12,13], validate their experiences [11], and enhance data-driven self-management [7,8,12], but only if the feedback is meaningful, and therefore, useful [14].

There is heterogeneity in the data visualization preferences of RMT users living with neurological and psychiatric conditions [15,16], but also some common overarching themes. Multiple studies have found that graphical data depictions [15,17], images, and color [16,17] enhance the RMT experience. A key principle is the generation of visualizations that are intuitive and clear [9,18], with a sensitivity to how users’ current health status affects their interests and ability to engage with RMT [19], making it clinically beneficial. Users have expressed a preference for data visualization that incentivizes and rewards positive health outcomes and behaviors, as opposed to providing negative feedback [16,20] and the ability to customize their visualization experience, for example, altering how often data is presented, and in what format and quantity it is visualized [12,21,22]. While user experience research has been conducted for pre-existing apps tailored toward the management of our 3 chosen relapsing conditions: multiple sclerosis (MS) [9], epilepsy [18], and depression [10,23], and has identified themes to consider in device selection and RMT design for individuals living with these conditions [19], none of these previous studies have focused specifically on data visualization. Existing studies focus broadly on multiple dimensions of service users’ design preferences and RMT user experiences (eg, health variables of perceived importance and perceived quality of medical advice provided) as opposed to data visualization preferences. This study was designed to address this gap. We chose to separate the 3 groups in terms of the participants’ diagnoses so that we could compare and contrast needs, and find common themes that were relevant transdiagnostically or different themes that were specific to each group.

A triangulation approach was adopted to integrate multiple methods of data collection and multiple sources of data [24]. This multimethod qualitative design facilitated the identification and validation of a thematic coding frame to improve our understanding of service users’ preferences and experiences of data visualization in RMT. Triangulation has been found to be an effective method for enhancing the credibility and validity of research findings [24,25] as synthesizing multiple data sources can help reduce bias and enrich data sets by broadening the scope and providing a more comprehensive picture [26,27]. This study builds on two sources of data (already published): (1) a systematic review [28], and (2) analyses of user reviews from existing self-management apps [29,30].

## Methods

### Overview

We ran several focus group sessions that allowed for the identification of new insights and an exploration of themes that had not been discovered in the previous data sources. This approach enabled a well-rounded exploration of visualization design preferences and permitted cross-validation of the thematic coding frame.

### Ethical Considerations

We sought and were granted ethical approval via the UK Health Research Authority’s research ethics committee (19/LO/1759). 

### Design of the Focus Groups

We recruited people with one of two neurological conditions: epilepsy or MS, or a diagnosis of major depressive disorder (MDD). Participants were eligible to participate if they met the following inclusion criteria; were at least 18 years old, fluent in English, able to provide informed consent, and had a clinical diagnosis of epilepsy, MS, or MDD (with an episode within the last 2 years). Participants with a confirmed diagnostic history of depression were recruited through the RADAR-CNS (Remote Assessment of Disease And Relapse - Central Nervous System) study [31], those with epilepsy and MS self-identified as having a diagnosis of one of these conditions and were recruited through nonprofit organizations (Epilepsy Action and MS Society) and were invited to join condition-specific focus groups.

### Contextual Measures

The following measures were completed to help characterize our samples of people with epilepsy, MS, or MDD: (1) demographic information (age, gender, ethnicity) was completed by all participants; (2) Patient Health Questionnaire [32] was completed by the MDD group. This is a validated 9-item measure of the severity of symptoms associated with MDD, with a threshold of ≥10 indicating current clinical problems; (3) Liverpool Seizure Severity Scale [33] was completed by the epilepsy group. It is a validated 20-item measure of patient-perceived seizure severity**,** and (4) Patient Determined Disease Steps scale [34] was completed by the MS group. It is a validated patient-reported outcome of disability in MS.

### Procedure

**Figure 1:**
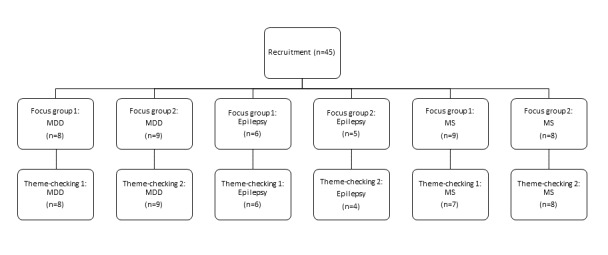
Flowchart of participants included in the focus groups and member checking sessions. MDD: major depressive disorder; MS: multiple sclerosis.

We held 12 focus groups, 4 for each health condition. Participants discussed data visualization with prompts from a topic guide (Multimedia Appendix 1). An initial analysis of the topics discussed in that focus group was sent to the participants who considered them and provided feedback in one of a further 6 “theme-checking” focus groups, which were held 2 weeks later (Figure 1). All questionnaires and consent forms were gathered by email and focus groups were conducted digitally, using videoconferencing software. The focus groups were recorded and transcribed prior to data analysis.

### Data Analysis

**Figure 2:**
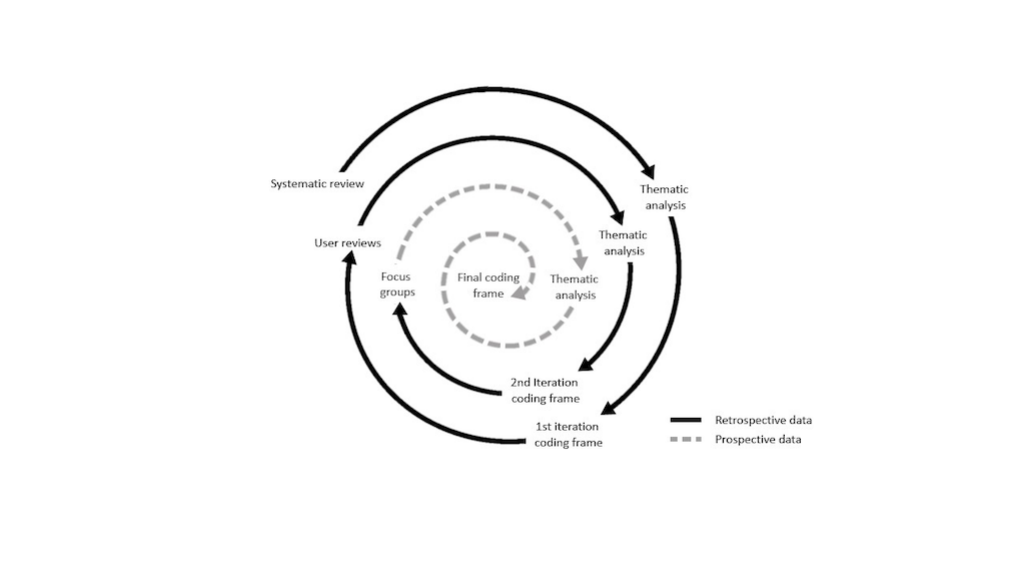
Coding frame development process.

The focus group discussions were shaped by a coding frame, which was developed through an iterative process, incorporating emerging themes from (1) the systematic review and (2) the analyses of user reviews for self-management apps (Figure 2). Focus group discussions were analyzed using a thematic approach. Content was analyzed deductively using the coding frame and refined inductively to reflect emerging themes. A total of 2 reviewers (ED-L and GG) coded the focus group transcripts and following coding, each reviewer suggested additions and revisions to the original coding frame. All disagreements were resolved through discussion. Reviews were then reread, and codes were compiled into descriptive themes. Novel themes emerging from the analysis were incorporated into the final version of the coding frame. The software NVivo (Lumivero) was used to help manage the data.

### Patient and Public Involvement

This research was codeveloped with an advisory board of people with lived experience of either depression, epilepsy, or MS who were gathered together to advise on the following program of study. The advisory board had oversight of the themes in the coding frame and helped with the interpretation of results. Representatives of this board are coauthors who have critically reviewed this paper.

## Results

### Focus Group Participant Characteristics

In total, 45 people were recruited to take part in the focus groups across the 3 health conditions (Figure 1). Participant characteristics are summarized in Table 1. People with epilepsy were mostly symptomatic at the time of participation (and rated themselves on average relatively high on severity), people in the depression group scored in the mild range but had a history of at least one episode of MDD in the last 2 years, and most people in the MS group reported a moderate level of ongoing disability.

**Table 1:** Participant characteristics (N=45).

Characteristic			Depression		Epilepsy		MS^a^		Overall
**Sex assigned at birth, n/n (%)**									
	Female	15/18 (83)		10/11 (91)		11/16 (69)		36/45 (80)	
	Male	3/18 (17)		1/11 (9)		5/16 (31)		9/45 (20)	
**Ethnicity, n/n (%)**									
	White or White British	15/18 (83)		11/11 (100)		16/16 (100)		42/45 (93)	
	Mixed	1/18 (6)		0/11 (0)		0/16 (0)		1/45 (2)	
	Other	2/18 (11)		0/11 (0)		0/16 (0)		2/45 (5)	
**Highest education completed, n/n (%)**									
	Degree level	14/18 (78)		6/10 (60)		10/16 (63)		30/44 (68)	
	A level	2/18 (11)		3/10 (30)		5/16 (31)		10/44 (23)	
	GSCE^b^ level	2/18 (11)		1/10 (10)		1/16 (6)		4/44 (9)	
Age (in years), n (mean, SD; range)			17 (55, 13; 27-69)		10 (41, 15; 23-62)		14 (50, 14; 33-73)		41 (50, 15; 23-73)
Age first diagnosed (in years), n (mean, SD; range)			17 (25, 12; 13-49)		10 (24, 16; 3-60)		14 (36, 11; 22-54)		41 (29, 13; 3-60)
**MS diagnosis, n/n (%)**									
	Relapsing remitting	0/0 (0)		0/0 (0)		13/16 (81)		—^c^	
	Primary progressive	0/0 (0)		0/0 (0)		1/16 (6)		—	
	Secondary progressive	0/0 (0)		0/0 (0)		2/16 (13)		—	
Symptom severity, n (mean, range)			17 (10.24, 2-17)		17 (57.35, 44.71)		10 (2.38, 0-6)		—

^a^MS: multiple sclerosis.

^b^GSCE: general certificate of secondary education.

^c^Not applicable.

### Thematic Coding Frame

**Figure 3:**
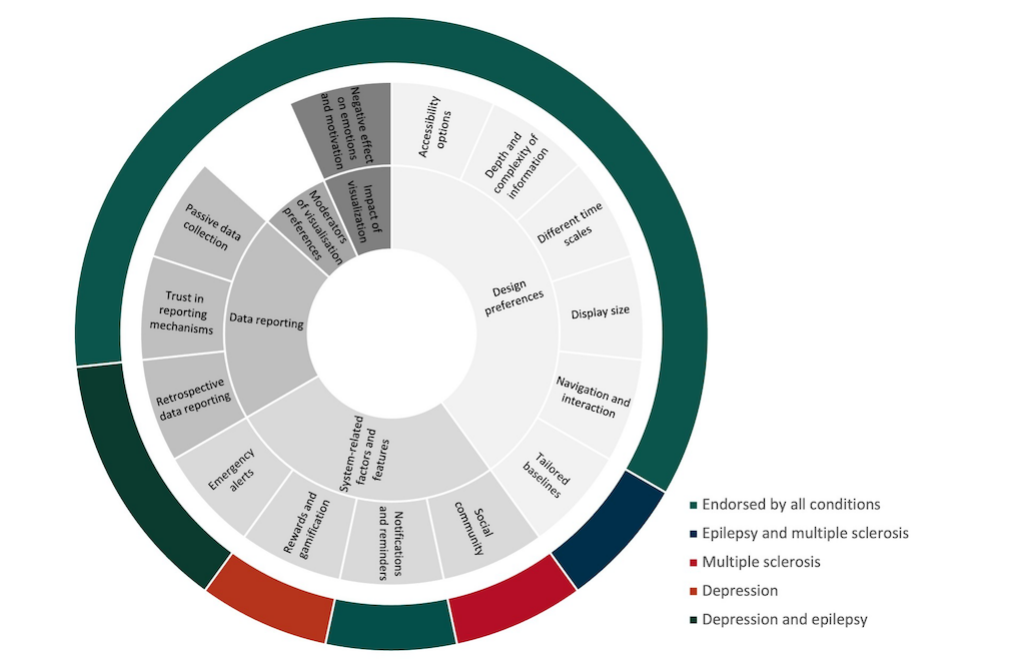
Visualization of the 14 novel subthemes and 4 expanded core themes.

In general, focus group discussions aligned with the first iteration of the coding frame for app-based health data visualization developed by Polhemus et al [28,29,30] (Table S1 in Multimedia Appendix 2). However, the focus groups yielded additional insights that had not been discussed in academic studies or app reviews, resulting in 14 novel subthemes and 4 expanded core themes (Figure 3). Despite the additions, the thematic structure of the coding frame remained unchanged, comprising five central themes: (1) design preferences and recommendations, (2) data reporting, (3) impact of visualization, (4) moderators of visualization preferences, and (5) system-related factors and features. Table S2 in Multimedia Appendix 2 provides a list of quotes that are illustrative of these themes.

### Major Theme: Design Preferences and Recommendations

As expected, this was the largest of all the themes emerging from the data. Bar and line graphs were the preferred format, regardless of diagnostic group, as they are easy to understand and can effectively visualize trends. All groups appreciated how numbers can help quantify experiences and that text can provide supplementary information (eg, contextual annotations). There was agreement within the groups that color influenced clarity and supported participants’ understanding of the data. Across conditions, the ability to manipulate the data display size was considered beneficial but was particularly critical for individuals with MS who often have visual impairments. For some, images, and the ability to upload photographs were thought useful. Members of the depression group thought animation might also be an engaging way of presenting data. The overarching sentiment across groups was that it was crucial for data to be simple and clear, with the option to view a greater depth and complexity of information, when required. All suggested that accessibility options, such as the ability to select color schemes and the availability of voice-activated software, were key to accommodating any visual and motor impairments. More generally, participants wanted a good user experience with ease of use, emphasizing straightforward navigation and interaction in the app.

All 3 groups expressed a desire for customization of data visualizations such as choosing app displays to suit their individual needs or interests. Participants also described the advantages of adaptive interfaces to select and present data of relevance to the user. For instance, when a user is experiencing an episode of depression, a seizure, or MS relapse, the app might respond by reducing visualizations of a decline in health or by increasing positive feedback through responses to behaviors captured in the app. People with MS were interested in the idea of having tailored baselines, which would ensure goals were realistic and that progress visualizations were motivating. All groups saw the value in being able to select, manipulate, and compare data streams, spotlighting the use of filtering data by different timescales (eg, daily, weekly, and monthly) to visualize immediate information and long-term trends.

### Minor Theme 1: Data Reported

**Figure 4:**
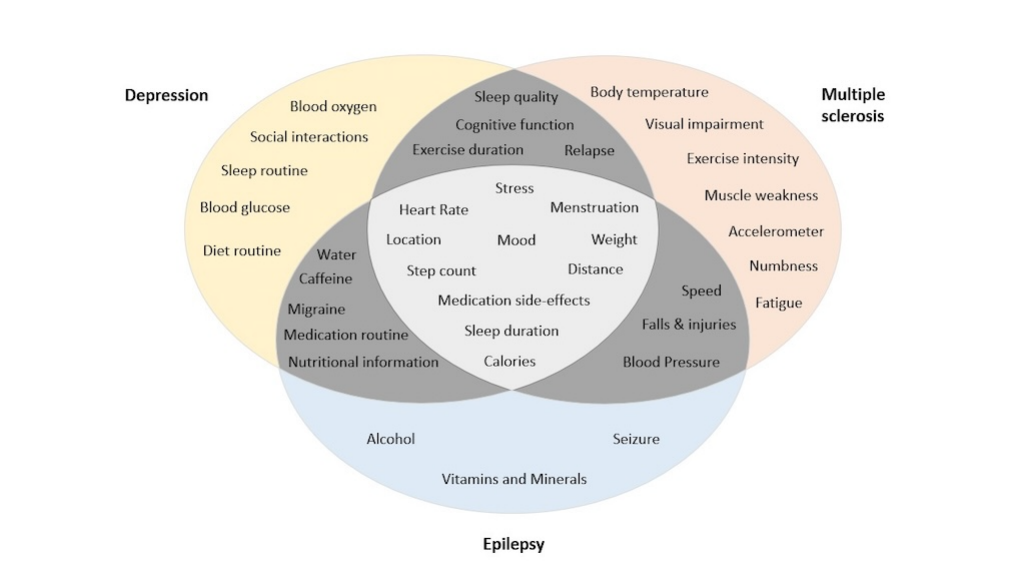
Independent and overlapping metrics of importance for the 3 diagnostic groups.

Participants from all 3 diagnostic groups highlighted additional metrics of importance that they agreed that they wished to track (Figure 4). Although there were condition-specific metrics, there were several intersecting measures that were important across conditions. The epilepsy and depression groups highlighted the need for a retrospective reporting capability, which allowed for flexibility around data input. The benefits of continuous, passive data collection were proposed by all groups. However, to maintain trust in reporting mechanisms, participants emphasized that data accuracy must be a top priority for RMT developers. These concerns were evident in instances where participants described experiences of apps that had collected data that was perceived to be inaccurate. Despite these concerns, participants still agreed on the benefits of translating subjective feelings (eg, mood) into “objective” or numeric data.

### Minor Theme 2: Impact of Visualization

Participants from all 3 diagnostic groups believed that data visualization would improve RMT engagement, participants felt that seeing their data would help to validate experiences, as well as structure and organize their symptoms, enabling proactive self-management. By identifying patterns and triggers, participants could become more self-aware of their health condition, effectively monitor progress, and possibly alter people’s experiences of their health condition. Another key impact was how data visualizations would affect an individual’s emotions and self-image. They could improve self-esteem by providing a sense of empowerment over their condition, on the other hand, negative data could lead to anxiety. All groups highlighted the potential of data visualizations to facilitate sharing and communication about their condition, particularly with health care professionals, but also government agencies and employers to validate and help accommodate their disability. This information could help family, friends, and carers be better informed, as well as members of the public (eg, in emergency situations such as a seizure).

### Minor Theme 3: Moderators of Visualization Preferences

This was the only theme in the coding frame not expanded by the focus group discussions, and all subthemes were mentioned across the 3 diagnostic groups. Participants described how their health status and intended use of the app affected their needs and requirements. Preferences were further moderated by experience with health monitoring, as it allowed participants to gain a better understanding of RMT and how best to use it.

### Minor Theme 4: System-Related Factors and Features

Participants had clear ideas about system-related factors and features. People in the depression and epilepsy groups wanted an app to provide visual emergency alerts if data indicated risk. More general “pop-up” notifications and reminders about upcoming appointments, medication, and positive behaviors (eg, exercise) were seen as beneficial by all diagnostic groups. Apps that used reward systems or gamification were considered a useful way of encouraging long-term engagement, especially in periods of low mood for people with depression. A social community feature would allow them to link with people with the same health condition and was seen as a benefit. Having the ability to export data was also seen as crucial for facilitating communication with health care practitioners.

## Discussion

### Principal Findings

This study provides the first cross-diagnostic analysis of data visualization preferences for RMT apps. Preferences only varied slightly between the conditions and visualization preferences coalesced under five broad themes (Table S1 in Multimedia Appendix 2). These themes corroborate and expand on our previous research looking at RMT user experience for individuals with epilepsy [18], MS [9,19], mental health conditions [10,12,15,17,21,23], and other neurological conditions [16,20,22], using a triangulation approach. We found that when asked directly, people had preferences for a combination of passive and active data collection that had not emerged in the previous data sources (ie, in our systematic review and analysis of app user reviews). A clearer emphasis on the importance of accuracy also arose. Some design preferences were unique, including discussions on the need for a mobile app to be accessible, with the size and complexity of the information displayed seen as key considerations to enable ease of navigation and interaction. While an interest in customization has been raised in the previous literature [35], the idea of using tailored symptom baselines, as well as flexible time scales, were novel insights from the focus group discussions. Participants expressed a desire for adaptive interfaces, which could use machine learning techniques to provide a more personalized user experience and feedback. Incentivization was considered important to support engagement with a mobile app, with new ideas around the use of gamification, social comparison, and the use of notifications emerging.

The impact of visualizations is such that health apps can enhance engagement and awareness, provide structure, and empower individuals to manage their conditions. Participants appreciate apps that are simple and intuitive but are simultaneously able to provide flexibility to accommodate individual health management needs. All diagnostic groups expressed a preference for pictures or graphics to display data and emphasized the need for clear formats with sufficient contextual information to allow users to navigate and understand their data. Overall, findings suggest that data visualizations can be an effective tool for health self-management when they align with users’ individual needs, interests, and goals. However, this is heavily reliant on the type of data that can be reported and the wider system that the app is placed within. These factors need to be considered in relation to data visualization preferences in future research.

### Limitations and Future Directions

The study design was a notable strength, as the triangulated framework enhanced validity and credibility, and the cross-diagnostic sample enhanced applicability and served as a point of comparison. The authors acknowledge that it was difficult to focus participants on the topic of data visualization, particularly as RMT design and data reporting are so intrinsically linked to data visualization. We recommend that future research studies adopt a more active approach to gathering individuals’ views on data visualization, for example, by asking participants to design their app display and then discuss their ideas. It should also be noted that where the topic guides in this study focused on the use of RMT for reviewing raw symptom data, an emerging subsection of the field is further exploring the potential for RMT data to predict deterioration or relapse [31]. Indeed, some participants mentioned the perceived value of emergency “risk” alerts within apps. Further work should look to understand how best to implement relapse prediction within data visualization components. Some individuals with depression had greater experience of using wearable devices, nonetheless, this transdiagnostic study detected few differences between the groups. However, examining data visualization preferences in a broader sample and recruiting from clinical services will ensure that these findings generalize to the wider population.

### Practical Implications and Conclusions

This study offers an opportunity for app designers to understand the data visualization preferences and priorities of individuals living with depression, epilepsy, and MS. The practical implications are considerable. RMT limits visualizations to mobile devices and introduces challenges related to scalability including reduced display sizes and resolution, lower computational power, and storage and interaction challenges associated with the smaller device size [14,36]. The need for simple visualizations matches with the current state-of-the-art data visualization style guide [36] and could be realized through a myriad of “out-of-the-box” solutions, for example, Google Charts. However, building in qualities, such as flexibility, intuitiveness, customization, and enhanced accessibility, requires increased development and resources. Similarly, considerable research efforts are still required to improve the generalizability and robustness of machine learning approaches within mobile health to provide a level of personalization that is reliable and supports clinical practice [14,37]. Together, these challenges point to the need for truly multidisciplinary co-design processes to meet patient preferences and priorities.

## Appendix

Multimedia Appendix 1 Focus group topic guide.

Multimedia Appendix 2 Additional tables.

## Abbreviations

MDD: major depressive disorder
MS: multiple sclerosis
RADAR-CNS: Remote Assessment of Disease And Relapse - Central Nervous System
RMT: remote measurement technology
