# Supplementary material for: Data Visualization Preferences in Remote Measurement Technology for Individuals Living With Depression, Epilepsy, and Multiple Sclerosis: Qualitative Study
Source: J Med Internet Res. 2024 Oct 18;26:e43954. doi: 10.2196/43954 (PMC11530729; doi:10.2196/43954)
Supplement: Multimedia Appendix 1 [file jmir_v26i1e43954_app1.docx]

**Topic 1: Previous experience with apps**

- Do you have any experience with using apps to monitor or manage your health?
- What were your reasons for using/not using apps?
- If yes: What data (i.e., symptoms, physical activity) was visualized in the apps? What did you like or dislike about the way information was visualized in the apps? How did the visualizations affect your experiences with the app?

**Topic 2: Finding out what data is of interest to you**

- What information about your health would you find useful to monitor and see displayed in an app?
- How do you think you would use that data? (Prompts: Some potential uses might be improving recall of symptoms, identifying/predicting triggers, communicating with others)
- Can you give me an example of a time when it would have been helpful to have a graph of your data?
- Is there any data you would not want displayed in an app? (why?)

**Topic 3: Preferences for seeing your data**

- Do you have any preferences on how you would like your data to be displayed?

(Prompt: Format, colors, line vs. pie chart, etc.)

- Show booklet of examples.
- Which ones do you like/dislike and why?
- Which examples are the clearest? The least clear?
- What are your thoughts on showing multiple types of data on the same graph? (i.e., multiple symptom scores, symptom scores and activities, etc.)
- Over what time period (day/week/month) should data be displayed?
- Do you have any other suggestions for the designers of these apps?

**Topic 4: Personalization**

- What kinds of situations might affect your data visualization preferences? (How might your preferences change in response to relapse, times of wellness, experience with tracking, early-stage vs. advanced disease, etc.)
- Are there any app features or contextual information you would wish to have when interpreting your data?

**Topic 5: Impact of seeing your data**

- What are the potential impacts of seeing your data in mobile apps? (These could be positive or negative) (examples could be: motivation, increased engagement, discouragement)
- Are there any strategies you would suggest to maximize the positive impacts?
- Are there any strategies you would suggest to minimize the negative impacts?
