# Supplementary material for: Data Visualization Preferences in Remote Measurement Technology for Individuals Living With Depression, Epilepsy, and Multiple Sclerosis: Qualitative Study
Source: J Med Internet Res. 2024 Oct 18;26:e43954. doi: 10.2196/43954 (PMC11530729; doi:10.2196/43954)
Supplement: Multimedia Appendix 2 [file jmir_v26i1e43954_app2.docx]

**Table S1.** Coding frame of all themes and subthemes, split by diagnostic group and data source.

| Major and minor themes | Subtheme (Level 1) | Subtheme (Level 2) | Systematic review | User reviews of existing apps | | | Focus groups | | |
| --- | --- | --- | --- | --- | --- | --- | --- | --- | --- |
|  |  |  |  | Depression | Epilepsy | MS | Depression | Epilepsy | MS |
| Design preferences and recommendations | User experience | Simplicity vs. complexity | X | X | X | X | X | X | X |
|  |  | Accessibility options |  |  |  |  | X | X | X |
|  |  | Navigation and interaction |  |  |  |  | X | X | X |
|  | Visualization format | Animation | X | X |  |  | X |  |  |
|  |  | Graphs | X | X | X | X | X | X | X |
|  |  | Images | X | X | X | X | X | X | X |
|  |  | Numbers | X | X | X | X | X | X | X |
|  |  | Text | X | X | X | X | X | X | X |
|  |  | Contextual annotation | X | X | X | X | X | X | X |
|  |  | Size |  |  |  |  | X | X | X |
|  |  | Colour | X | X | X | X | X | X | X |
|  |  | Shape | X | X |  |  |  |  |  |
|  |  | Depth and complexity of information |  |  |  |  | X | X | X |
|  | Customization | Selecting, manipulating, and comparing data streams | X | X | X | X | X | X | X |
|  |  | Individual needs and interests | X | X | X | X | X | X | X |
|  |  | Adaptive interface | X | X | X | X | X | X | X |
|  |  | Tailored baseline |  |  |  |  |  |  | X |
|  | Timeliness of data access and visualization | Timeliness | X | X | X | X | X | X | X |
|  |  | Different time scales |  |  |  |  | X | X | X |
| Data reported | Metrics of importance |  |  | X | X | X | X | X | X |
|  | Passive data collection |  |  |  |  |  | X | X | X |
|  | Retrospective reporting |  |  |  |  |  | X | X |  |
|  | Accuracy of data |  | X | X | X | X | X | X | X |
|  | Trust in reporting mechanisms |  |  |  |  |  | X | X | X |
|  | Benefits of translating subjective feelings into objective data |  | X | X |  |  | X | X | X |
|  | Optional vs. Compulsory |  |  | X | X | X |  |  |  |
|  | Identifying with data reporting mechanisms |  | X | X |  |  |  |  |  |
| Impact of visualization | Affecting self-image |  | X | X | X | X | X | X | X |
|  | Engagement with RMT |  | X | X | X | X | X | X | X |
|  | Enabling proactive self-management |  | X | X | X | X | X | X | X |
|  | Increased self-awareness | Identifying patterns and triggers | X | X | X | X | X | X | X |
|  |  | Monitoring progress | X | X | X | X | X | X | X |
|  | Altering symptoms |  | X | X | X | X | X | X | X |
|  | Improve recall of past experiences |  | X | X | X | X |  |  | X |
|  | Validating current feelings and experiences |  | X | X | X | X | X | X | X |
|  | Providing structure and organisation |  | X | X | X | X | X | X | X |
|  | Negative effect on emotions and motivation |  |  |  |  |  | X | X | X |
|  | Sharing and communication | Caregivers | X |  | X | X | X | X | X |
|  |  | Healthcare professionals | X | X | X | X | X | X | X |
|  |  | Public | X | X |  |  | X | X | X |
| Moderators of visualization preferences | Context/Intended use |  | X | X | X | X | X | X | X |
|  | Experience with health monitoring |  | X | X | X | X | X | X | X |
|  | Health status |  | X | X | X | X | X | X | X |
|  | Personality |  | X |  |  |  | X | X | X |
| System-related factors and features | Developer response to feedback |  |  | X | X | X |  |  |  |
|  | Technical Issues |  |  | X | X | X |  |  |  |
|  | Rewards and Gamification |  |  |  |  |  | X |  |  |
|  | Emergency alerts |  |  |  |  |  | X | X |  |
|  | Notifications and reminders |  |  |  |  |  | X | X | X |
|  | Social community and support |  |  |  |  |  |  | X | X |
|  | Generating and Exporting Reports |  |  | X | X | X | X | X | X |
|  | Connectivity/ Interoperability |  |  | X | X | X |  |  |  |
|  | Cost, Finance, or Paywalls |  |  | X | X | X |  |  |  |
|  | Other Feature or Functions |  |  | X | X | X |  |  |  |
| New subthemes identified in focus group discussions in grey | | | | | | | | | |

**Table S2.** Illustrative quotes for some of the themes and subthemes that emerged during the data visualization focus groups, split by diagnostic group.

| Diagnostic group (participant) | Theme | Subtheme(s) | Quote |
| --- | --- | --- | --- |
| Epilepsy (P8; Group 1) | Visualization Format | Types of data visualization → Text | It's [text] just less interesting to look at than having some kind of clear graph, having something visual makes it a lot clearer and easier to understand. |
| Epilepsy (P39; Group 2) | Visualization Format | Types of data visualization → Images | It would be useful to have a way of perhaps uploading photos to an app that gives a bit more information of where the seizure happened and what kind of damage may have been done. |
| Epilepsy (P8; Group 1) | Visualization Format | Format Characteristics → Colour | If there's multiple types of data, to make it clearer, like what each, which type of data each is referring to, like if it was each different colour or something, um, that would be quite helpful |
| Epilepsy (P13; Group 1) | Design Preferences and Priorities | Features and Functions → Emergency system | I think with a sort of seizure button, one of the things that I would say is very, very useful is if you have that [medical history] readily accessible, because whether it’s paramedics, whether it’s the partner, family member or friend, or even in yourself if you’re in a postictal state, if it’s very complex to reach that information, it may not be the priority, whereas I think sometimes it’s much more helpful if whoever is aiding you or if you need to get aid yourself, you can access the information that something’s happened very, very quickly. |
| Epilepsy (P13; Group 1) | Design Preferences and Priorities | Features and Functions → System-related factors → Export data into a report | I think as part of the reporting process it would be good if the app could communicate either with your GP or your neurologist, or whoever your sort of first port of all is within the NHS ... if they can almost get the report as soon as a seizure has happened, then at least the information is there and they know - you don't have to spend time explaining it to them during that limited time frame you have. |
| Epilepsy (P11; Group 2) | Data reported | Retrospective reporting | [Following a seizure] I struggle ... to speak and all I wanna do is sleep, and I sleep for probably near enough the clock round. Um, I don't wanna do anything. I can't really engage very well, so it would be a waste of time to even try to operate the app, text me or remind me to engage in a conversation. I just sleep. So, it would be something that I would have to do once I was back in the land of the living, shall we say. |
| Epilepsy (P13; Group 1) | Data reported | Benefits of translating subjective feelings into objective data | I think for me, one of the big things will be moving away from sort of the more speculative side, and the more subjective side of assessing what the patterns are around seizures and actually being able to have some immediately available objective data that can then help you to understand what just happened, but more importantly understand what the triggers are moving forward, that that would be a massive positive. |
| Epilepsy (P34 Group 1) | Impact of Visualization | Increase self-awareness → Identifying patterns and triggers | Having the objective data and then and being able to look to see what triggers there might be that you haven't perhaps been able to be aware of before because you're recording, because the app’s recording lots of information, it might point to something that you’ve- subtly is there that you've not been aware of. |
| Multiple sclerosis (P36; Group 1) | Customization | Adaptive interface → Tailored baselines | I think it's really important that this is tailorable… that the app is tailoring goals and targets to an individual’s baseline, based on intelligent programming that observes your patterns over time, rather than a pre-established ‘normal’… |
| Multiple sclerosis (P42; Group 1) | Design Preferences and Priorities | Features and Functions → Social community | I think communicating trends with other people with MS would be interesting. So, if somebody said to me: “you're having walking difficulties, I follow this sort of pattern”, and they've done a piece of analysis, it would then be good if I could replicate their analysis on myself and say, actually I'm having the same sort of problems that person and then you could kind of work with them in a subgroup to try and work out management strategies and say: “well that worked for them, maybe it'll work for me”, if you can find a common pattern between you. |
| Multiple sclerosis (P26; Group 1) | Design Recommendations | Accessibility Options | What you've got to bear in mind, I mean, it's very well documented stuff about app development for colours and zoom[ing in on visualizations] and stuff like that, but the difference for people with MS is that you're gonna have some people who are gonna have manual dexterity problems, as well as people who've got cognitive problems or visual problems, so it's how the standard rules of design need to be adapted for our needs. Because I, for example, find it very difficult with my fingers to expand on the telephone screen. |
| Multiple sclerosis (P35; Group 2) | Design Recommendations | Ease of Use | I just think that we've got to be a little bit mindful that we're not sort of trying to overcomplicate everything to one in five apps or one in four apps people abandoned after a day. You need to make something that is simple and is going to agree with people… |
| Multiple sclerosis (P1; Group 1) | Impact of Visualization | Improve recall of past experiences | We might forget, you know how many falls we've had or when we had them. Sometimes we forget how tired we were on certain days. So, if you're looking back and thinking yeah, um, you know looking at the data you might not have remembered that on Wednesday, it was a really bad day, fatigue wise. |
| Multiple sclerosis (P33; Group 2) | Impact of Visualization | Increase self-awareness → Monitor progress | To be able to look back over the last month, and realize that although I can only walk, you know, that although I can only walk down to the shops and back today over the month, I’ve been better at walking then I was the month before, just looking at a little bit of positive reinforcement. |
| Depression (P20; Group 2) | Visualization Format | Types of data visualization → Graphs and charts | Personally, I prefer bar charts or line charts because they are quick and easy... I think a pie chart does not necessarily represent the information particularly well…if you can see it going up and down, your highs and lows (e.g., bar and line charts), that makes it much easier to understand and comprehend. |
| Depression (P29; Group 1) | Visualization Format | Types of data visualization → Animation | I would personally love something animated and show you the weeks progress in real time. So, you could watch as it moved across the screen, so you can see it how your sleep was going up and down. I find that really interesting… at the end of the week we could look at the week's progress and see… how it rises and falls. |
| Depression (P3; Group 1) | Visualization Format | Format Characteristics → Depth and complexity of information | It’s all about having as much information as possible in any way, and whether I can choose to look at it at a higher level or to dig down deeper into the detail. I'd like to have it all available if I want it… I like to have the headlines first and then when I've got the time or I want to, to be able to dig down into the detail. |
| Depression (P16; Group 1) | Timeliness | Retrospective → Different time scales | I like a choice of time scales because sometimes I want to see just today or this week, but I also want to have a look at the long-term trends. |
| Depression (P38; Group 1) | Customization | Selecting, manipulating, and comparing data streams | A search function or even a filter for…manipulating your data, so you can see exactly what you'd like to at that moment. |
| Depression (P12; Group 1) | Design Preferences and Priorities | Features and Functions → Emergency alerts | I’d really like something which flags up that your mood is slipping… it would be so useful to have something which sort of basically says “just hang on, you're really, you know, going downhill fast… if your sleep goes down or you're eating increases then that could be used surely to give a warning that you're at risk of going downhill with depression or something. |
| Depression (P15; Group 1) | Design Preferences and Priorities | Features and Functions → Rewards and gamification | If there was some way building in some kind of reward system where I'm not quite sure what the reward would be, but something some additional motivation, to sort of engaged with it… just something that would make people they would get people hooked on it, I suppose… so to use it more and to make use of it. And maybe with the times were not feeling so good, or so motivated. |
| Depression (P29; Group 1) | Design Recommendations | Navigation and Interaction | I would like the ability to hold your phone one way and get the simple view and then just turn up your phone and get the more detail. For me, that would be a lot easier because if I'm having a bad day and my hands are cramping to not have to press too much but just flip the phone and get what I need, that would be really good…and there could almost be a menu of things that can be displayed, and then you choose the ones you want from that menu and then they will be displayed. |
| Depression (P17; Group 2) | Impact of Visualization | Enable proactive self-management | If you get feedback from your wearable or from an app, it might be that you are then able to manage that and say: “oh, hang on what happened at this specific time?” and “I know this has triggered me in the past, what am I going to do to manage it better?... once we've been given the data, we then know how to use it to feel better about ourselves, which is kind of proof that the apps do you work, you can see the cause, and then you can then say: “ah, this has happened, so now I know I need to do this. |
